# Pharmacogenomic-Guided Prescribing for Treatment-Resistant Mental Health Conditions in Australian Primary Care: A Single-GP Practice Retrospective Observation of 29 Patients

**DOI:** 10.3390/ijms27167329

**Published:** 2026-08-17

**Authors:** Cristina Beer, Fiona Rae, Mikayla Watt, Annalese Semmler, Joanne Voisey

**Affiliations:** 1 Centre for Genomics and Personalised Health, School of Biomedical Sciences, Faculty of Health, Queensland University of Technology, Kelvin Grove, QLD 4059, Australia; cristina.beer@hdr.qut.edu.au (C.B.); f.rae@qut.edu.au (F.R.); 2 Dr. Cris Medical Services, Robina, QLD 4226, Australia; mikaylagwatt@gmail.com; 3 School of Clinical Sciences, Faculty of Health, Queensland University of Technology, Kelvin Grove, QLD 4059, Australia; annalese.semmler@qut.edu.au

**Keywords:** pharmacogenetics, psychotropic drugs, primary health care, precision medicine, cytochrome P-450 enzyme system

## Abstract

Treatment-resistant mental health conditions are common in primary care and challenging for clinicians. Trial-and-error prescribing can prolong morbidity and increase adverse drug reactions (ADRs). Pharmacogenomic (PGx) testing enables individualised prescribing by identifying gene–drug interactions affecting psychotropic response. Thirty adults with treatment-resistant mental health conditions underwent PGx testing using a commercial panel (one lost to follow-up [*n* = 29]). Patients received PGx-guided treatment (*n* = 8) or standard care (*n* = 21). Phenotypes were assigned per CPIC and DPWG guidelines, with prescribing guided by clinical experience where guidelines were unavailable. Medication histories were reviewed for gene–drug concordance, ADRs, and treatment failures. Clinical improvement at eight weeks was defined as “marked” or “moderate” improvement and/or ADR resolution. Actionable genotypes were common, particularly CYP2D6 (27.5% poor/intermediate drug metabolising phenotype) and CYP2C19 (37.9%). Guideline-actionable gene–drug interactions occurred in 37% of patients, and eleven patients possessed actionable phenotype at multiple loci. Gene–drug interactions were identified in nine patients and guidance was fully implemented in six. Clinical benefit at 8 weeks was achieved in 6/8 patients with genotype-guided changes versus 8/21 receiving standard care. PGx-guided prescribing may support improved antidepressant response and tolerability while reducing trial-and-error prescribing for treatment-resistant patients in primary care.

## 1. Introduction

Mental health disorders remain one of the leading contributors to global disease burden and disability [1]. Pharmacotherapy is a cornerstone of treatment across depressive, anxiety, bipolar and psychotic illnesses [2]. Yet, despite the proliferation of antidepressants and other psychotropics, clinical response rates remain modest. The process of identifying an effective and tolerable medication often depends on an empirical “trial-and-error” approach, exposing patients to prolonged morbidity and adverse drug reactions (ADRs). Large-scale studies have repeatedly highlighted the striking inter-individual variability in treatment response [3]. In 2023–2024, general practitioners (GPs) initiated approximately 84% of all psychotropic prescriptions in Australia, underscoring the importance of practical strategies that can improve prescribing precision within primary care [4].

Over the past decade, PGx has evolved from research to clinical implementation, supported by international guideline consortia such as the Clinical Pharmacogenetics Implementation Consortium (CPIC) and the Dutch Pharmacogenetics Working Group (DPWG), and more recently as a user-friendly platform known as ClinPGx [5]. In Australia, the Royal College of Pathologists of Australasia (RCPA) has contextualised these international frameworks for clinical application. In 2024, the RCPA released national comprehensive PGx testing guidelines for 35 commonly used drugs, including several antidepressants and/or antipsychotics, with the aim to improve treatment efficacy and safety [6]. These guidelines, aligned with CPIC and DPWG frameworks, represent the primary reference for clinical settings. Furthermore, a recent publication evaluated the use of the 35 medications indicated in the RCPA PGx recommendations and found potential clinical benefit for implementing PGx testing into routine practice [7].

Pharmacokinetic heterogeneity contributes substantially to non-response and ADR risk. Cytochrome P450 (CYP) enzymes, particularly CYP2D6, CYP2C19, CYP1A2 and CYP2B6, catalyse phase-I oxidation of most antidepressants, antipsychotics, mood stabilisers and psychostimulants [8]. Despite multiple psychotropic options, response rates remain modest. Large observational cohort studies report 30–50% discontinuation of first-line antidepressants due to inadequate efficacy or ADRs [9]. Even when second- or third-line agents are trialled, cumulative remission rates in naturalistic settings rarely exceed 60%. The Sequenced Treatment Alternatives to Relieve Depression (STAR*D study), frequently cited as the definitive real-world effectiveness trial, required four sequential treatment steps to achieve a 67% cumulative remission rate; each switch was accompanied by progressively lower response probabilities and higher dropout from ADRs [10]. A recent real-world case study of 40 patients with ineffectiveness or ADRs to antidepressants reported a high prevalence of actionable CYP2D6 and CYP2C19 variants and clinically meaningful improvements after PGx-guided prescribing, further supporting the utility of PGx testing in difficult-to-treat populations [11].

The practical implementation of PGx in mental health care is supported by accumulating clinical evidence. Multiple systematic reviews and meta-analyses confirm that PGx-guided prescribing of antidepressants improves remission odds, reduces ADRs and decreases the number of medication changes required to achieve response [12]. The GUIDED trial (*n* > 1100) found combinatorial PGx reporting achieved 28% greater remission than standard care [13]. Subsequent meta-analyses replicated these findings across heterogeneous study populations and laboratory platforms. Economic evaluations by the RCPA estimate that Australian implementation could save over $1 billion per year by implementing PGx testing and thereby avoiding ADRs [14].

Despite the maturation of the evidence base, most antidepressant prescribing in Australia remains guided by empirical symptom response rather than genetic profiling. Australia’s government-funded healthcare system, Medicare, does not provide an item number for multigene PGx testing, limiting equitable access. Several private health fund organisations are now offering reimbursement for PGx testing in Australia,, albeit awareness amongst patients and general practitioners remains low. Implementation has largely been confined to tertiary psychiatry services and academic research programmes [15]. Nevertheless, the increasing availability of clinical-grade laboratory panels and educational resources positions primary care for wider adoption. GPs already manage most psychotropic prescribing and are uniquely placed to integrate PGx testing into medication reviews and shared decision-making.

In summary, PGx represents a practical avenue to move prescribing from empirical trial-and-error to evidence-based precision therapy. Given that Australian GPs are responsible for most psychotropic prescribing, evaluating multigene PGx testing in primary care provides essential evidence to inform national policy, guide education and support equitable patient access. The present study therefore aimed to determine the frequency of actionable PGx variants among Australian patients with treatment-resistant mental health conditions and assess whether genotype-guided prescribing improved clinical outcomes. This study aims to determine whether PGx testing of key CYP enzymes (CYP2D6, CYP2C19, CYP2C9, CYP3A4, CYP1A2, and CYP2B6) in adults with treatment-resistant or difficult-to-treat mental health conditions improves psychotropic selection, tolerability, and clinical outcomes in primary care.

## 2. Results

### 2.1. Patient Characteristics

For full demographics see Supplementary Table S1; 29 patients were included (65.5% female, 90% Caucasian, *M* = 46.6, *SD* = 16.2, range 21–89). Two patients were of East-Asian descent, and one from mixed descent. A total of 24% of patients were ex-smokers (*n* = 7) and 13.8% hazardous alcohol users (AUDIT-C ≥ 4). Primary psychiatric diagnoses: 11 major depression (38%), 11 generalised anxiety (38%), four ADHD/ADD (13.8%), three autism-spectrum disorder (10.3%), two bipolar-II (6.9%), and one schizophrenia (3.45%). Ten met formal treatment-resistant depression criteria (≥2 failed adequate courses of antidepressants). Physical comorbidities included hypertension (41.4%), dyslipidaemia (34.5%), gastro-oesophageal reflux (27.6%), and chronic pain (27.6%). Biochemistry revealed six patients with stage-2 chronic-kidney disease, six with ALT/AST > 1.5× upper limit and five with Gilbert-related unconjugated hyperbilirubinaemia. During the 8-week follow-up period, 15/29 patients had documented concurrent psychotherapy involvement.

### 2.2. Genotypes and Phenotypes

Based on genotypes, phenotype subgroups were allocated as displayed in Supplementary Table S2: 27.59% of patients were CYP2D6 PM or IM (two PM, six IM); 37.93% were CYP2C19 PM or IM (one PM, ten IM); 37.93% CYP1A2 UM (*n* = 11). Three of 27 genotyped for *CYP2B6* carried reduced-function alleles (*6/*6 or *4/*6). Nine patients (31.04%) were CYP2C9 IM or PM (eight IM, one PM), and two (6.9%) were CYP3A4 IMs. The rare CYP3A4*22 variant appeared once. The remainder were normal metabolisers for each enzyme. Eleven patients possessed actionable phenotypes at multiple loci.

### 2.3. Historical Psychotropic Exposure and Adverse Events

Patients had tried a median of three distinct agents (range 1–7). Table 1 summarises the psychotropics previously trialled by patients included in the current cohort, grouped by class, and the associated primary CYP metabolic pathways. Common ADRs precipitating cessation included excessive sedation (8/29), gastrointestinal upset (6/29), increased anxiety (5/29) and sexual dysfunction (4/29).

### 2.4. Actionable Gene–Drug Interactions

Nine patients (31%) exhibited at least one CPIC/DPWG level A/B actionable gene–drug pair. Table 2 presents the actionable gene–drug interactions, including the number of cases and corresponding prescribing recommendations.

The GP fully adopted guidance in six cases (switches *n* = 4, dose reductions *n* = 2); advice was partially implemented in one (dose increased but agent retained) and declined in two. Nine CYP2C9 IM/PM carriers and two CYP3A4 IMs generated no traffic-light alerts because psychotropic-specific dosing tables are unavailable for these loci [16,17,18]. Any potential impact on clinical outcomes is speculative and based on literature review. For example, CYP2C19 and CYP2C9 have minimal influence on agomelatine metabolism [19], and CYP1A2 variation alone is no longer considered sufficient to guide olanzapine dosing (DPWG, 2023 update; [20]). These analyses are descriptive only.

### 2.5. Eight-Week Clinical Outcomes

Twenty-nine patients presented for follow-up to assess for potential improvement after an 8-week period (one lost to follow-up). Clinical improvement was assessed approximately 8 weeks after the PGx-guided medication adjustment, though some variability occurred due to differing return times of PGx reports. Among nine with fully/partially implemented recommendations, eight had evaluable data (one lost). Table 3 demonstrates the degree of clinical improvement in these eight patients and the associated medication changes.

Overall, six of eight patients who achieved genotype-guided changes achieved clinical benefit. In the standard-care group, eight of 21 achieved clinical benefit. These results are descriptive and exploratory due to the small sample size.

## 3. Discussion

This retrospective observational cohort study demonstrated that multigene PGx testing undertaken in a single general-practice setting identified guideline-actionable *CYP* variants in 37% (*n* = 11) of patients with treatment-resistant mental health conditions. GP-implemented genotype-concordant changes resulted in 75% (*n* = 6) of these patients showing substantial clinician-rated improvement or complete ADR resolution within 8 weeks. Although statistically under-powered, the direction and magnitude of benefit are consistent with evidence from larger psychiatric-clinic trials showing improved antidepressant response and tolerability when treatment is guided by PGx testing [12,13]. Interpreted solely from the laboratory report without external decision support these findings suggest minimal-infrastructure PGx can add tangible clinical value in Australian primary care.

The actionability rate mirrors the Genomics Used to Improve Depression Decisions (GUIDED) trial, which found 33% of patients had actionable gene––drug interactions and PGx-guided treatment yielded a 28% higher remission rate compared with usual care [13]. Similarly, meta-analyses of randomised controlled PGx trials consistently show that PGx-guided antidepressant prescribing improves treatment response and remission rates in mental health [12,13]. The current study differs from these investigations conducted in specialist settings with multidisciplinary interpretation teams. By contrast, this study was performed in a single GP clinic without external decision-support infrastructure. The GP applied the PGx colour-coded guidance unaided and achieved similar relative benefit. Although preliminary, these findings are consistent with larger studies and suggest clinically meaningful benefits may be achievable by integrating PGx into Australian general practice.

Biologically, all observed clinical improvements had a plausible pharmacokinetic foundation. CYP2D6 PMs switched from venlafaxine to desvenlafaxine bypassed the need for CYP2D6-dependent O-demethylation, decreasing the occurrence of ADRs. Dose escalation of escitalopram in CYP2C19 UMs resulted in faster metabolic clearance. Likewise, CYP2D6 IMs who received a reduced paroxetine dose experienced fewer serotonergic ADRs. For an equivalent dose, PMs may experience five- to ten-fold greater drug exposure than UMs, resulting in adverse effects in the former and subtherapeutic response in the latter. It is important to emphasise that genotype represents only one determinant of a patient’s phenotypic response. Non-genetic factors, such as concomitant medications, alcohol use, and other environmental exposures, also contribute significantly and must be considered when interpreting overall phenotype. This underscores a key implementation lesson: genotype must be interpreted in the biochemical and pharmacological context in which it operates [8].

Although *CYP2C9* and *CYP3A4* variants were not formally “actionable,” their prevalence (30% and 6.7%, respectively [*n* = 9 and 2]) prompts two considerations. First, subtle exposure differences may explain residual non-response or ADRs that persisted despite CYP2D6/CYP2C19 optimisation. Second, forthcoming CPIC and DPWG updates are expected to cover agomelatine, diazepam and atypical antipsychotics whose kinetics depend on these loci.

The present findings add to the growing international evidence base supporting PGx implementation beyond tertiary care. The United Kingdom’s National Health Service (NHS) Genomic Medicine Service recently launched national PGx pilots within primary care, offering CYP2D6/CYP2C19-guided antidepressant prescribing in general-practice settings [21]. Early outcomes show high clinician satisfaction, reduced medication switches, and improved treatment adherence. Similarly, Singapore’s Ministry of Health has incorporated PGx into its National Precision Medicine strategy, embedding testing within polyclinics and public hospitals, where it guides prescribing of antidepressants and/or antipsychotics [22]. In the United States, the Veterans Affairs system introduced nationwide PGx testing in 2021, integrating results into electronic health records for real-time decision support across mental health services [23].

Collectively, these initiatives demonstrate that PGx-guided psychotropic prescribing is scalable, cost-effective, and compatible with primary care workflows when supported by clear reporting frameworks, standardised guidelines, and clinician education. The present study extends this global progress by providing local Australian data showing that meaningful outcomes can be achieved using a minimal-infrastructure model guided by laboratory reports alone. The findings of this single GP clinic suggest preliminary Australian primary care data supporting the feasibility of PGx-guided prescribing in everyday general practice.

The frequent criticism of PGx testing in clinical practice is that the interpretation adds cognitive load and prolongs consultation time. Emerging evidence, particularly from paediatric and adolescent mental health cohorts, suggests pre-emptive PGx testing may offer greater clinical benefit compared with testing after treatment failure [24,25], highlighting the potential value of integrating PGx earlier in treatment pathways. The GP reported only an average of six extra minutes per result disclosure, less than the mean eight minutes added by integrating cardiovascular absolute-risk charts into routine reviews. No external pharmacist or genetic counsellor was engaged, although larger practices may benefit from such support. Vendor training comprised a single two-hour webinar, suggesting that primary care-focused educational packages can achieve rapid competency uptake. Other barriers to clinical integration include the lack of Medicare reimbursement for PGx testing [15], minimal formal training for GPs, and variable awareness of interpretation frameworks.

Ethnic variability influences clinical application. Allele frequencies differ significantly between ancestral groups, affecting the proportion of patients in each phenotype category. For instance, *CYP2C19* loss-of-function alleles are present in up to 30% of East-Asian individuals compared with about 15% in Europeans [26]. Such differences imply that PGx findings derived primarily from European cohorts may not directly extrapolate to diverse populations. Australia’s multicultural demography therefore necessitates inclusion of Aboriginal, Torres Strait Islander, Asian and African ancestries in national allele-frequency databases to ensure equitable interpretation of PGx results.

Consistent with previous studies exploring the substantial differences in genetic variability in *CYP2C19* across ethnic or ancestral groups, the two East-Asian patients in the current study carried *CYP2C19* loss-of-function alleles (*2/*2* and *2/*3*) [26]. These findings are consistent with previous data exploring how this affects drug metabolism and response in individuals with different ethnic backgrounds [26]. Therefore, the findings in predominately European cohorts may not be directly applicable to populations with different genetic backgrounds. The sample was mostly Caucasian, reflecting inner-metropolitan Queensland demographics, and PGx testing costs were privately borne. Health-policy initiatives that subsidise testing for high-need groups may ameliorate disparities while generating cost-offsets through avoided ADR-related consultations.

### 3.1. Strengths and Limitations

Strengths of this study include the minimal-infrastructure model, consideration of biochemical modifiers and inclusion of neurodevelopmental disorders. Limitations include small sample size and the reliance on clinician judgement to categorise clinical improvement (e.g., marked or moderate improvement) without the use of validated psychiatric outcome measures or patient-reported outcome measures. This introduces the potential for observer bias and limits the objectivity and reproducibility of the findings. Additional limitations include phenotypic lag, incomplete gene coverage (only known variants were assessed) and lack of objective adherence metrics. As only patients consenting to PGx testing were included, findings may reflect a self-selected cohort and may not generalise to all patients with treatment-resistant mental health conditions.

Although not discussed in detail in this paper, nearly half of the cohort used omega-3 or L-methylfolate and 10% (*n* = 3) used St John’s wort, agents known to interact with CYP enzymes or neurotransmitter systems. Although no severe herb––drug interactions were recorded, hyperforin-rich St John’s wort induces CYP3A4 and P-glycoprotein, potentially lowering quetiapine or mirtazapine levels [27]. The myDNA report did not automatically account for such nutraceuticals, leaving interaction assessment to clinical judgement. Future iterations of laboratory reports should incorporate commonly encountered complementary medicines to mitigate this gap. Another limitation is that PGx prescribing guidelines are not updated frequently enough to reflect emerging evidence. Although no formal prescribing recommendations currently exist for some genes, they were included in the GP PGx report and are therefore noted here for completeness.

Despite these constraints, the study offers a valuable proof-of-concept for GP integration of PGx testing. It provides local data supporting the broader international consensus that multi-gene PGx testing enhances psychotropic safety and efficacy.

### 3.2. Clinical Implications

This study supports practical guidance for PGx-guided prescribing in primary care. The cohort included patients with ≥2 prior psychotropic trials; however, broader PGx guidance suggests consideration of testing after the first failed antidepressant trial to accelerate optimisation [12,13]. Core genes remain CYP2D6 and CYP2C19, with CYP1A2 for smokers, CYP2B6 for sertraline, and CYP2C9/CYP3A4 for selected agents. Interpretation can be achieved via straightforward laboratory reports, though Clinical Decision Support tools may enhance accessibility and scalability. Patient engagement is facilitated by traffic light coding and fostering shared decision-making but may create expectancy effects.

### 3.3. Future Research

Priority directions comprise cluster-randomised trials comparing PGx-guided versus usual-care prescribing, using validated psychiatric outcome measures and patient-reported outcome measures to objectively assess treatment response, economic modelling using Australian Medicare data, equity studies in diverse communities and AI-enhanced clinical decision support (CDS) that integrates genotype, real-time laboratory data and prescribing history.

Future large-scale, controlled studies, integrating cost-utility analyses and assessing diverse populations, are essential to validate these results and to inform equitable reimbursement strategies, but the present data provides preliminary proof-of-concept data that implementation of genetics-guided prescribing is feasible and ready for mainstream adoption in Australia.

## 4. Methods and Materials

### 4.1. Study Design, Setting and Ethical Considerations

A retrospective observational cohort study was conducted at a single-GP metropolitan clinic in Queensland, Australia, offering integrative mental health services. The practice introduced optional PGx testing in July 2022 for patients with difficult-to-treat psychiatric presentations. The study window spanned July 2022 to July 2024. All eligible patients during this period were included; no a priori sample-size calculation was performed as the analysis constituted a complete case-series review. As a preliminary study, this was exploratory in nature and aimed to understand implementation feasibility, generate preliminary clinical observations, and inform the design of future research. The Queensland University of Technology Human Research Ethics Committee approved the study (Project 091-HE09).

### 4.2. Patients

Electronic medical records were screened to determine inclusion: completion of PGx testing; adults ≥ 18 years; DSM-5 diagnosis of mental health condition; at least two previous psychotropic trials discontinued or dose-reduced due to non-response (<25% clinical improvement after ≥6 weeks at guideline dose) or ADR (e.g., intolerable sedation, sexual dysfunction, QT prolongation). This definition was applied across psychiatric diagnoses, as the cohort included depressive, anxiety, neurodevelopmental, bipolar and psychotic disorders. Exclusion criteria: Unstable mental health requiring hospitalisation at time of sampling (*n* = 1). In total, 30 patients initially met all inclusion criteria, of whom 29 completed follow-up assessments. The standard-care group included patients with no actionable variants or those who declined actionable recommendations. As neither received PGx-guided prescribing, these groups were combined due to small sample sizes. Baseline characteristics were comparable, though differences in disease course cannot be fully excluded.

### 4.3. Laboratory Procedures and Report Structure

Laboratory testing was performed as part of routine clinical care and not specifically for this study; results were extracted for analysis. Genomic DNA was isolated from self-collected buccal swabs and processed in the National Association of Testing Authorities, Australia (NATA)-accredited myDNA laboratory (Melbourne, Australia) within 7–10 days of receipt. Genotyping of *CYP2D6*, *CYP2C19*, *CYP2C9*, *CYP3A4*, *CYP1A2*, and *CYP2B6* was performed on a custom Infinium^®^ microarray (Illumina Inc., San Diego, CA, USA), and copy-number variation at *CYP2D6* (*5 deletion; gene duplications) was confirmed with reflex TaqMan^®^ quantitative PCR. The panel interrogated clinically actionable alleles, *CYP2D6* *2, *3, *4, *5, *6, *7, *8, *9, *10, *14A, *14B, *17, *29, *36, *39, *41 and gene duplications; *CYP2C19* *2, *3, *17; *CYP2C9* *2, *3; *CYP3A4* *22; *CYP1A2* *1F; and *CYP2B6* *4, *6, with the reference *1 allele inferred when no variant was detected. Diplotypes were auto-called and translated to metabolic phenotypes (poor, intermediate, normal, rapid, ultrarapid) using current activity-score tables from CPIC and DPWG, then manually reviewed by clinical geneticists and pharmacists before report release.

### 4.4. Reporting and Guideline Framework

Pharmacogenomics (PGx) investigates how inherited genetic variation affects drug disposition and response, enabling genotype-guided precision prescribing. Clinical PGx testing assigns diplotypes for key pharmacogenes, most commonly cytochrome P450 (CYP) enzymes, and translates these to predicted metabolic phenotypes. Individuals with a normal (extensive) metaboliser (EM) phenotype are expected to have reference CYP activity and typical dose requirements. Altered drug metabolising phenotypes reflect a change in enzyme function and are categorised as poor (PM), intermediate (IM), rapid (RM), and ultrarapid (UM) metabolisers, which guide drug selection, dosing, and monitoring [16].

Each PGx report used a traffic-light format (green = standard dosing acceptable, orange = caution: dose adjustment or more frequent monitoring suggested, red = consider alternative agent) with hyperlinks to CPIC and DPWG guidance. Recommendations included examples such as dose reduction for *CYP2D6* PMs on venlafaxine or avoidance of clozapine in heavy smokers who are *CYP1A2* UMs. Potential drug–drug and drug–nutrient interactions were flagged qualitatively. When PGx guidelines were unavailable or inconclusive, prescribing decisions were guided by clinician experience and pharmacological knowledge.

### 4.5. GP Education and Workflow

The practice’s mental health lead GP (author) attended a two-hour myDNA webinar on report interpretation. No external pharmacist, genetic counsellor or electronic decision-support system was used. During routine follow-up (median 22 days post-sample), the GP reviewed the colour-coded report with each patient, explained genotype implications, and proposed management (dose reduction, switch, discontinuation, or no change). Implementation was documented in the electronic medical record as: Implemented—all report recommendations adopted; Partially implemented—dose altered but agent retained, or switch delayed; Declined—GP or patient opted not to change therapy.

### 4.6. Data Collection

The following data were manually extracted from the electronic medical record: demographics, body-mass index (BMI), smoking status, alcohol use; comorbidities coded by Charlson Comorbidity Index (CCI); baseline laboratory data: estimated Glomerular Filtration Rate (eGFR) (CKD-EPI), Alanine Aminotransferase (ALT), Aspartate Aminotransferase (AST), γ-glutamyl transferase (GGT), unconjugated bilirubin; complete medication list, including over-the-counter supplements; historical psychotropic exposure (name, dose, duration, cessation reason); baseline DSM-5-oriented narrative; presence of ADRs; follow-up clinician assessment at 8 weeks categorised a priori as “marked”, “moderate”, “minimal” or “no improvement”; as well as ADR status as “resolved”, “persisting”, or “new”. Medication adherence was assessed using available documentation from patient self-report, prescription refill history, and clinician assessment recorded in consultation notes. Engagement in concomitant psychological therapies was also extracted from clinical records. Narrative entries were double-coded by two researchers. Discrepancies were reconciled by consensus. No ICD-10 or SNOMED diagnostic codes were used; exposures and outcomes were derived from narrative clinical notes, so no code list applies.

### 4.7. Bias Control and Data Quality Assurance

To minimise selection and information bias, all keyword-identified records were manually reviewed by two researchers to confirm eligibility. Duplicate entries were removed, and variable ranges were screened for implausible values before import into the analysis dataset. The electronic medical record platform was Best Practice (version 1.13.0.1061), which maintains immutable audit trails. The GP-author held full read-only privileges, while extractors held analyst-level access in accordance with institutional data-governance policies.

### 4.8. Outcome Definitions

An actionable gene–drug interaction is defined as the presence of a CPIC or DPWG level A/B recommendation affecting a psychotropic the patient had ever used. Clinical benefit was determined at 8 weeks. Resolution of the ADR was categorised by a GP as “marked” when symptoms no longer met thresholds for clinical significance and functional impairment was absent or minimal (consistent with remission, informed by DSM-5 criteria), and as “moderate” when clinically meaningful improvement was observed but residual symptoms and/or functional impairment persisted.

## 5. Conclusions

In this single-GP, retrospective observational cohort, multigene PGx testing identified clinically actionable *CYP* variants in 37% (*n* = 11) of difficult-to-treat patients and, when acted upon, was associated with a 75% (*n* = 6) response of meaningful symptom relief or complete ADR resolution within eight weeks. These preliminary findings suggest that precision psychopharmacology can be delivered in Australian primary care with minimal additional infrastructure, offering a practical alternative to the longstanding trial-and-error approach.

The feasibility of this model requiring only a single educational session and minimal additional consultation time provides a strong foundation for future research evaluating the broader implementation of PGx-guided prescribing in Australian general practice. Broader adoption will depend on Medicare funding, education of clinicians, integration into electronic medical records, and inclusion of diverse ethnic populations in Australian allele-frequency databases. If supported by larger prospective studies, PGx testing has the potential to reduce trial-and-error prescribing, improve treatment adherence, and deliver measurable cost savings to the Australian health system. These findings align with the RCPA’s vision for genomics-informed healthcare and suggest that precision psychopharmacology may be feasible in a single GP setting.

## Figures and Tables

**Table 1:** Psychotropics previously trialled and major CYP pathways.

	Class	Medication	Primary Pathway(s) *
Antidepressants	Selective serotonin reuptake inhibitor (SSRI)	Citalopram, Escitalopram	CYP2C19
		Sertraline	CYP2C19
		Paroxetine	CYP2D6
	Serotonin–noradrenaline reuptake inhibitor (SNRI)	Venlafaxine	CYP2D6
		Duloxetine	CYP2D6, CYP1A2
	Noradrenergic and specific serotonergic antidepressant (NaSSA)	Mirtazapine	CYP3A4
		Mianserin	CYP3A4
	Melatonergic antidepressant/atypical	Agomelatine	CYP2C19 **
	Tricyclic antidepressant (TCA)	Amitriptyline	CYP2D6, CYP2C19
		Nortriptyline	CYP2D6
Antipsychotics	Atypical antipsychotic	Olanzapine	CYP1A2
		Risperidone	CYP2D6
	Mood-stabilising atypical antipsychotic	Quetiapine	CYP3A4
Stimulant	Central nervous system stimulant	Dexamphetamine	CYP2D6
Drugs for Anxiety	Benzodiazepine anxiolytic	Diazepam	CYP2C19, CYP3A4

* Metabolic pathways are mainly derived from CPIC and DPWG guidance [8,16]. ** Here, the enzyme is shown in italics because while they clearly contribute to agomelatine’s biotransformation, no CPIC or DPWG dose-adjustment guidance yet exists for this drug. Legend CYP, cytochrome P450 enzyme; GABA, gamma-aminobutyric acid.

**Table 2:** Actionable pharmacogenomic combinations.

Genotype and Drug	Cases (*n*)	Recommended Action
CYP2D6 Poor Metaboliser + Venlafaxine	4	Switch to desvenlafaxine or duloxetine
CYP2C19 Ultra-Rapid Metaboliser + Escitalopram	2	Increase dose by 50% or switch to alternative
CYP2D6 Intermediate Metaboliser + Paroxetine	2	Reduce dose by 25% or switch to alternative
CYP2B6 Decreased Function + Sertraline	1	Flagged with yellow “caution”

**Table 3:** Impact of genotype-guided treatment on patient outcomes.

Outcome	Number of Participants and Frequency %	Notes
Marked improvement	3 (38%)	2 venlafaxine PMs switched to desvenlafaxine; 1 paroxetine IM dose reduced
Moderate improvement	3 (38%)	1 escitalopram UM dose escalated by 50%; 2 mirtazapine yellow cautions prompted bedtime timing
Minimal/No change	2 (25%)	Both had elevated GGT (>120 IU/L)

Legend: PM, poor metaboliser; IM, intermediate metaboliser; UM, ultrarapid metaboliser; GGT, gamma-glutamyl transferase. Note: Resolution of ADRs: night sweats (*n* = 1), visual blurring (*n* = 1). No new ADRs emerged after genotype-driven modifications. ADR was categorised as marked when symptoms no longer met DSM-5 diagnostic thresholds and functional impairment was absent or minimal, and was moderate when clinically meaningful improvement occurred but residual symptoms and/or functional impact persisted.

## Data Availability

The raw data supporting the conclusions of this article will be made available by authors on request.

## Supplementary Materials

The following supporting information can be downloaded at: https://www.mdpi.com/article/10.3390/ijms27167329/s1.
