# Supplementary material for: Pharmacogenomic-Guided Prescribing for Treatment-Resistant Mental Health Conditions in Australian Primary Care: A Single-GP Practice Retrospective Observation of 29 Patients
Source: Int J Mol Sci. 2026 Aug 17;27(16):7329. doi: 10.3390/ijms27167329 (PMC13513532; doi:10.3390/ijms27167329)
Supplement: Supplementary file 1 [file ijms-27-07329-s001.zip › ijms-4459685-supplementary.pdf]

Table S1.

Summary demographics and baseline characteristics of cohort

Characteristic		n (%)
**Sample size, n**		29
**Age, years, mean ± SD, range**		46.6 ± 16.2 (21–89)
**Gender**	Female	19 (65.52)
**Ethnicity**	Caucasian/European	26 (89.66)
	East Asian	2 (6.9)
	Other/Mixed	1 (3.45)
**Primary mental health diagnosis**	Depression	11 (37.93)
	Anxiety	11 (37.93)
	ADHD/ADD	4 (13.79)
	ASD	3 (10.34)
	Bipolar-II	2 (6.9)
	Schizophrenia	1 (3.45)
**Mental-health comorbidities**	Anxiety	17 (58.62)
	Depression	10 (34.48)
	Sleep disorder	7 (24.14)
	Post-natal depression	5 (17.24)
	PMDD	3 (10.34)
	PTSD	2 (6.9)
**Physical comorbidities**	Hypertension	12 (41.38)
	Dyslipidaemia	10 (34.48)
	GORD	8 (27.59)
	Chronic pain	8 (27.59)
	Type 2 diabetes	6 (20.69)
	Obesity (BMI ≥30)	5 (17.24)
	Asthma/COPD	4 (13.79)
	Osteoarthritis	4 (13.79)
**Alcohol use (standard drinks/week)**	0	15 (51.72)
	1–3	3 (10.34)
	4–6	5 (17.24)
	7–10	2 (6.9)
	>10	4 (13.79)
**Smoking status**	Current	0
	Ex-smoker	7 (24.14)

**Total current medications**	0	19 (65.52)
	1	7 (24.14)
	2	2 (6.9)
	3	1 (3.45)
**Psychotropic medications trialled**	0	13 (44.83)
	1	12 (41.38)
	2	2 (6.9)
	3	1 (3.45)
	4	1 (3.45)
**Total nutraceuticals in use**	0	4 (13.79)
	1	4 (13.79)
	3	3 (10.34)
	4	2 (6.9)
	5	9 (31.03)
	6	5 (17.24)
	7	2 (6.9)
	11	1 (3.45)
**Laboratory abnormalities**	Abnormal hepatic	6/25 (24.0)
	Abnormal renal (eGFR <90)	6/24 (25.0)
	Elevated bilirubin	5/25 (20.0)

Legend. ADHD, Attention Deficit Hyperactivity Disorder; ADD, Attention Deficit Disorder; ASD, Autism Spectrum Disorder; GORD, Gastro-Oesophageal Reflux Disease; PMDD, Premenstrual Dysphoric Disorder; PTSD, Post-Traumatic Stress Disorder; eGFR, estimated Glomerular Filtration Rate; BMI, Body Mass Index; SD, Standard Deviation.

Table S2.

Cohort Pharmacogenetic (PGx) Phenotypes

Genotype	Phenotype	N (%)
CYP2D6 phenotype (n=29), n (%)		
	PM	2 (6.9)
	IM	6 (20.69)
	NM	21 (72.41)
CYP2C19 phenotype (n=29), n (%)		
	PM	1 (3.45)
	IM	10 (34.48)
	NM	5 (17.24)
	RM	11 (37.93)
	UM	2 (6.9)
CYP2C9 phenotype (n=29), n (%)		
	PM	1 (3.45)
	IM	8 (27.59)
	NM	20 (68.97)
CYP1A2 phenotype (n=29), n (%)		
	PM	0
	IM	0
	NM	17 (58.62)
	RM	1 (3.45)
	UM	11 (37.93)
CYP3A4 phenotype (n=29), n (%)		
	PM	0
	IM	2 (6.9)
	NM	27 (93.1)
CYP2B6 phenotype (n=27), n (%)		
	PM	3 (11.11)
	IM	11 (40.74)
	NM	11 (40.74)
	RM	2 (7.41)
	UM	0

Note: Table S2 includes both guideline-actionable (CPIC/DPWG level A/B) and non-guideline CYP findings; only level A/B interactions should be interpreted for clinical decision-making.

Legend. PM, poor metaboliser; IM, intermediate metaboliser; NM, normal metaboliser; RM, rapid metaboliser; UM, ultrarapid metaboliser.
